# Supplementary figures and images for: Loss of polycystins suppresses deciliation via the activation of the centrosomal integrity pathway
Source: Life Sci Alliance. 2020 Jul 10;3(9):e202000750. doi: 10.26508/lsa.202000750 (PMC7368097; doi:10.26508/lsa.202000750)

# Figure S2

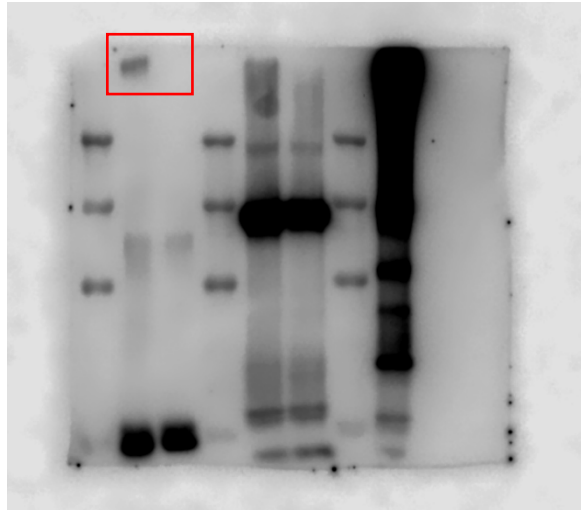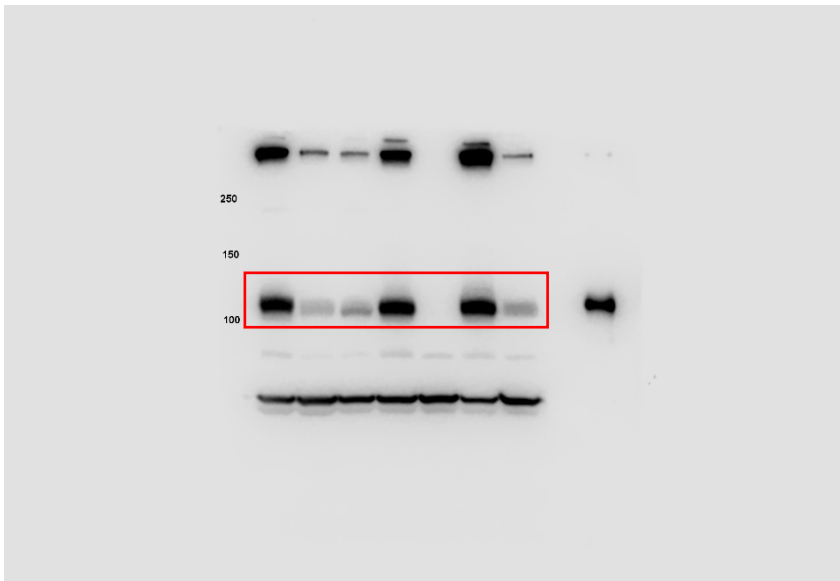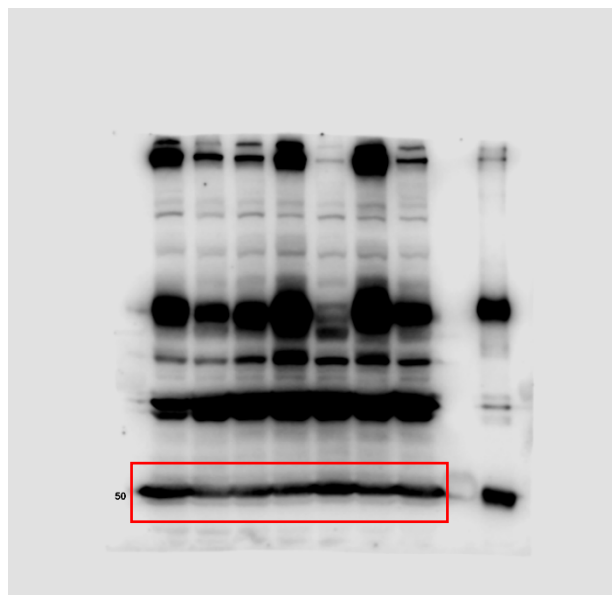

Supplement: Supplementary file 1 [file LSA-2020-00750_SdataFS2.pdf]

# Figure 3B

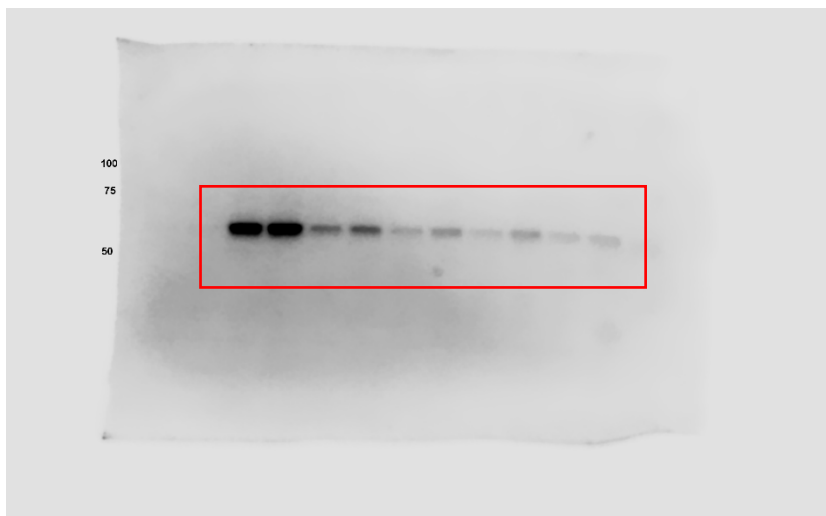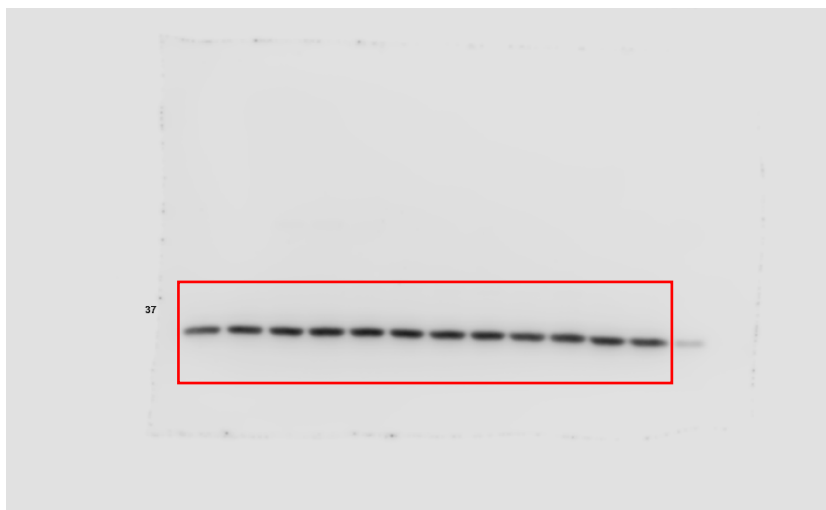

Supplement: Supplementary file 2 [file LSA-2020-00750_SdataF3.pdf]

# Figure 4A

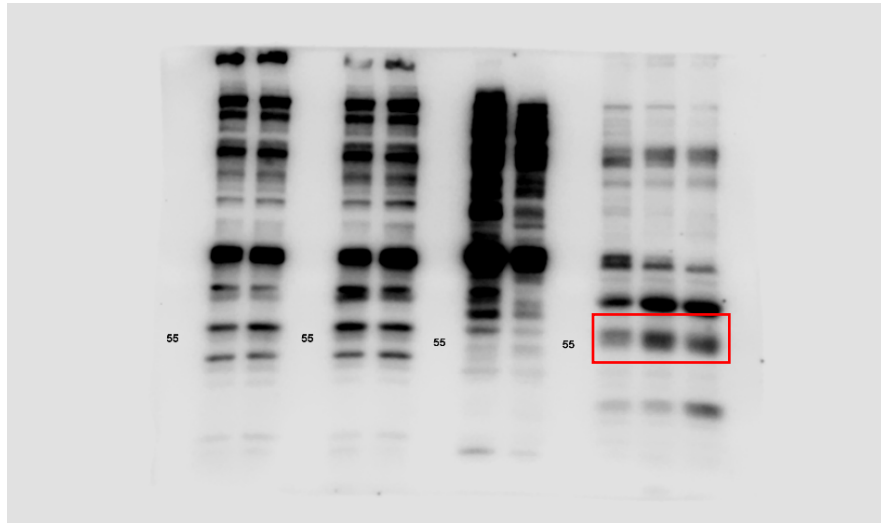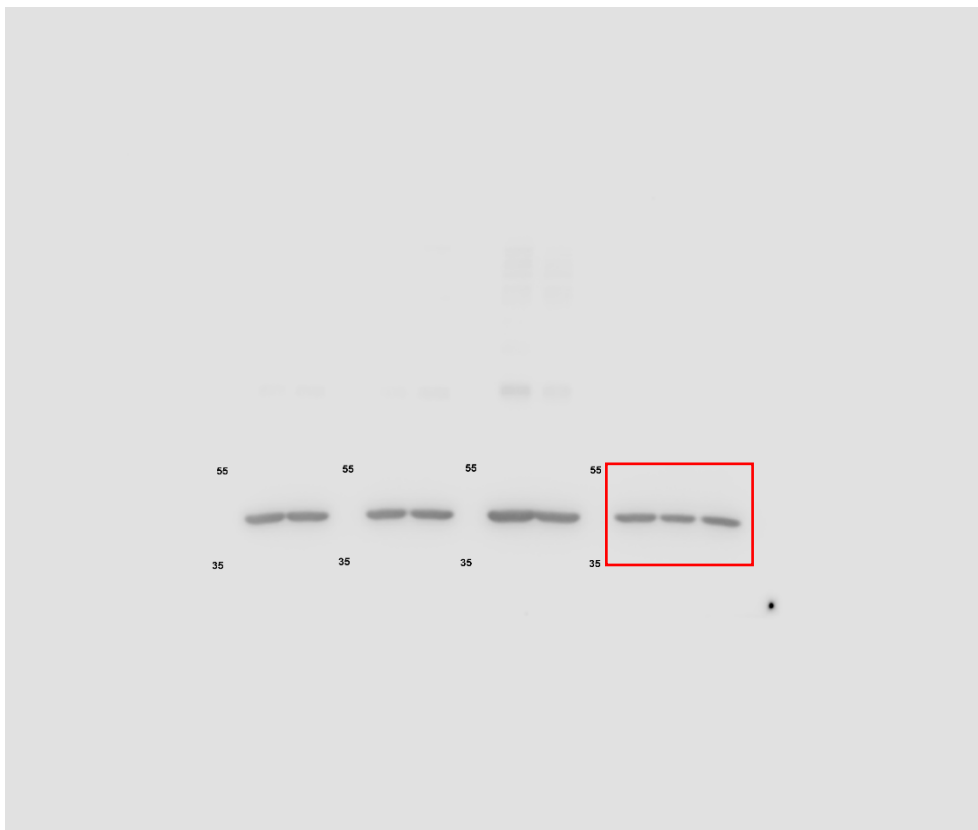

# Figure 4B

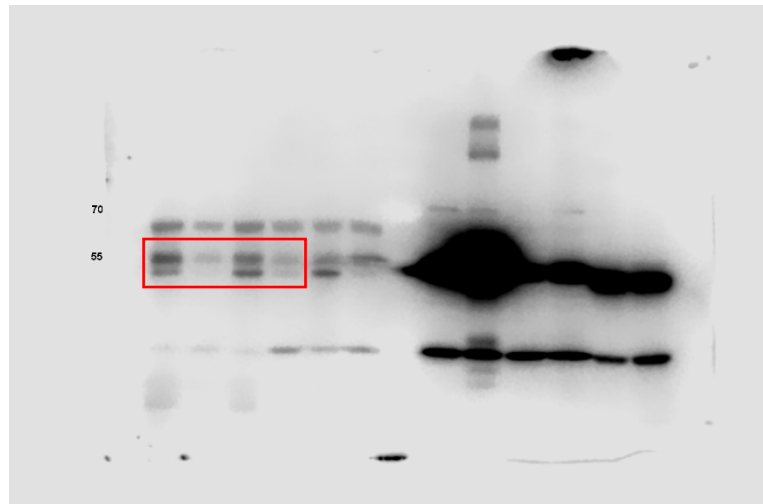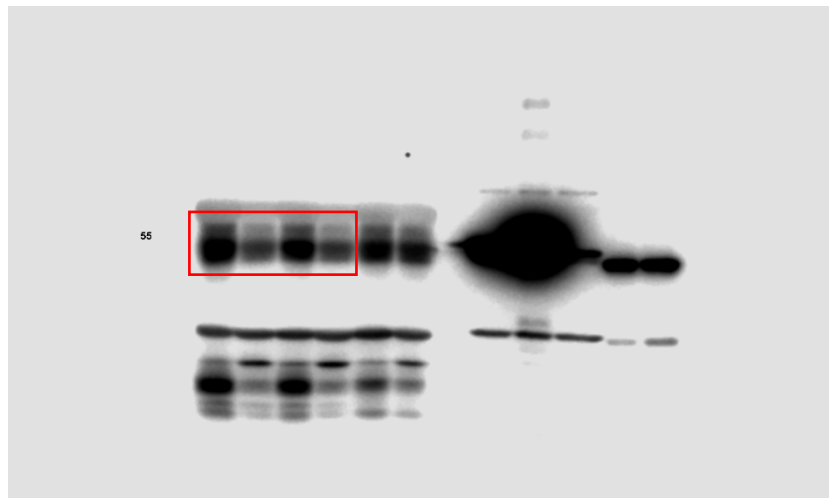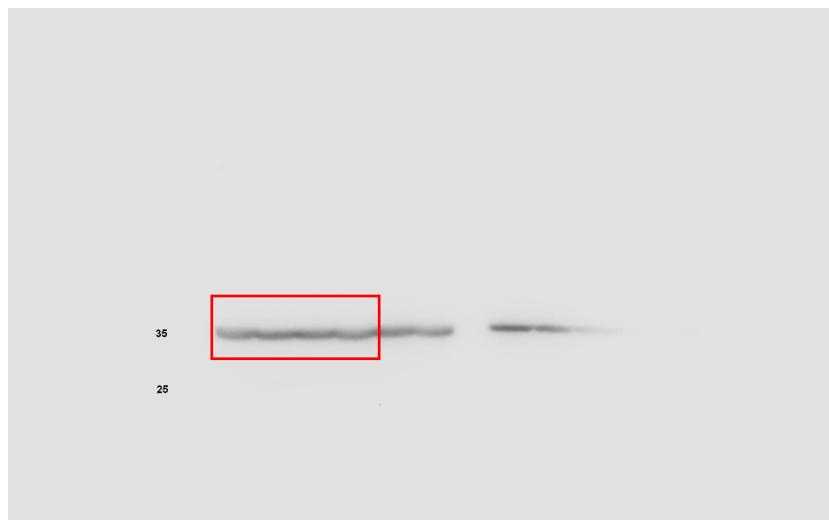

Supplement: Supplementary file 3 [file LSA-2020-00750_SdataF4.pdf]

# Figure S3

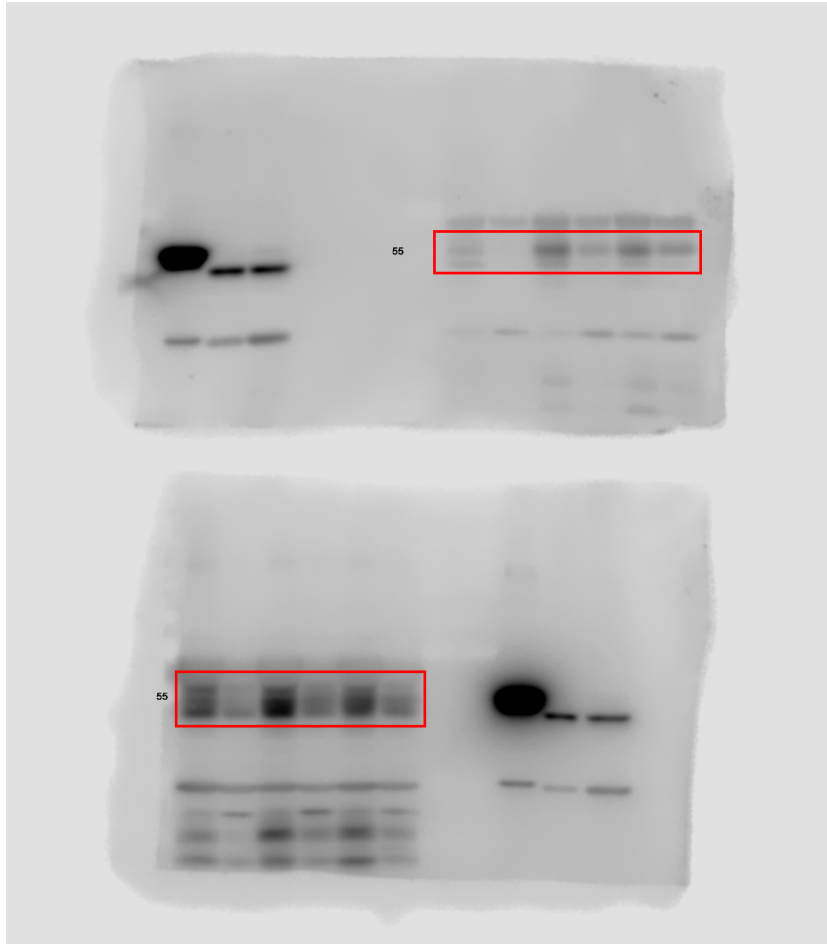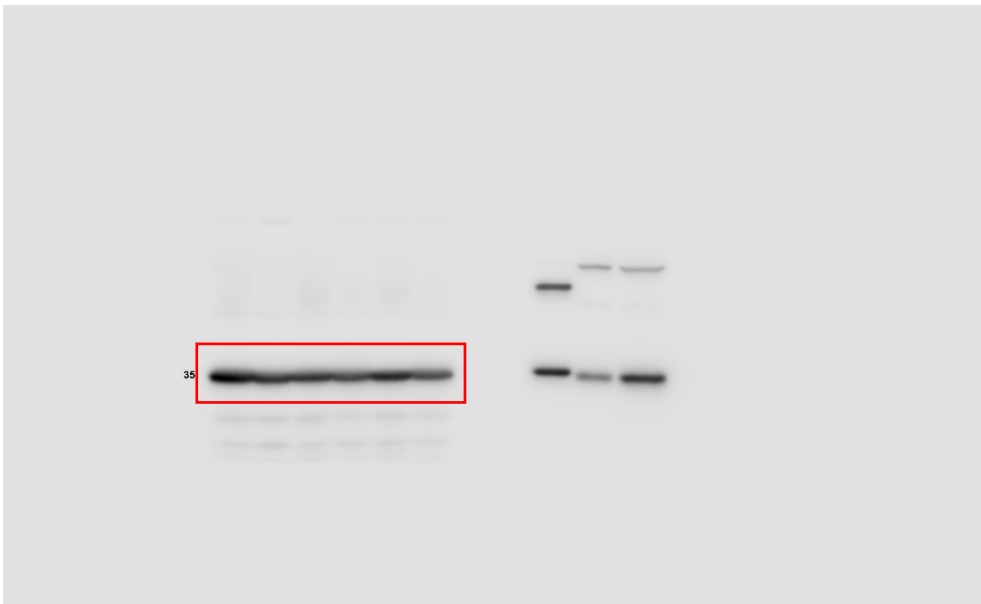

Supplement: Supplementary file 4 [file LSA-2020-00750_SdataFS3.pdf]

# Figure S6

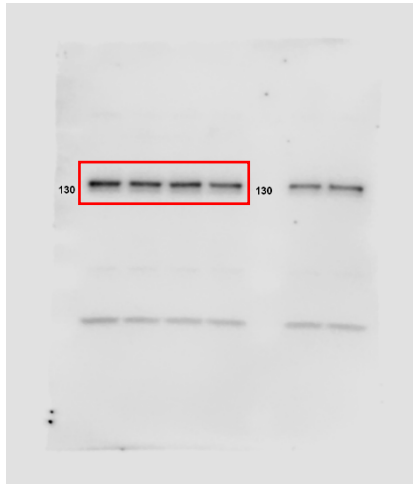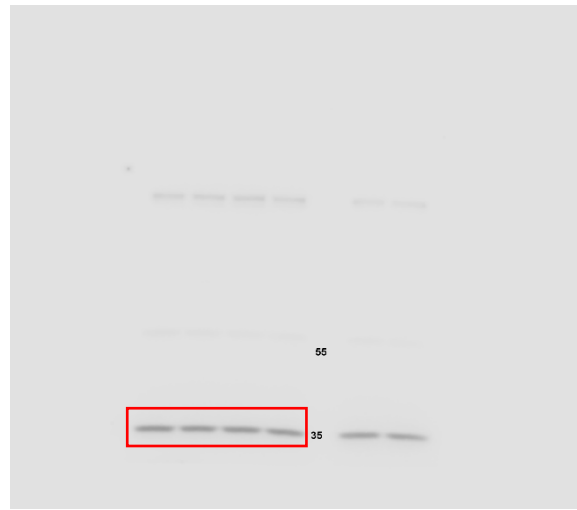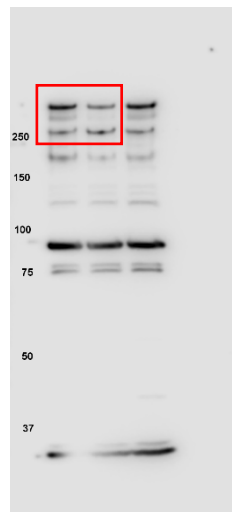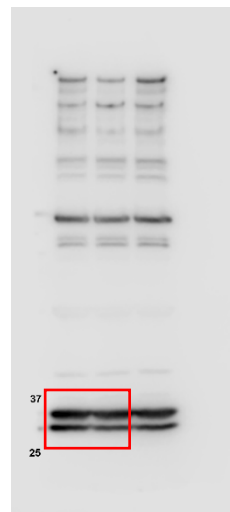

Supplement: Supplementary file 5 [file LSA-2020-00750_SdataFS6.pdf]

# Figure 6A

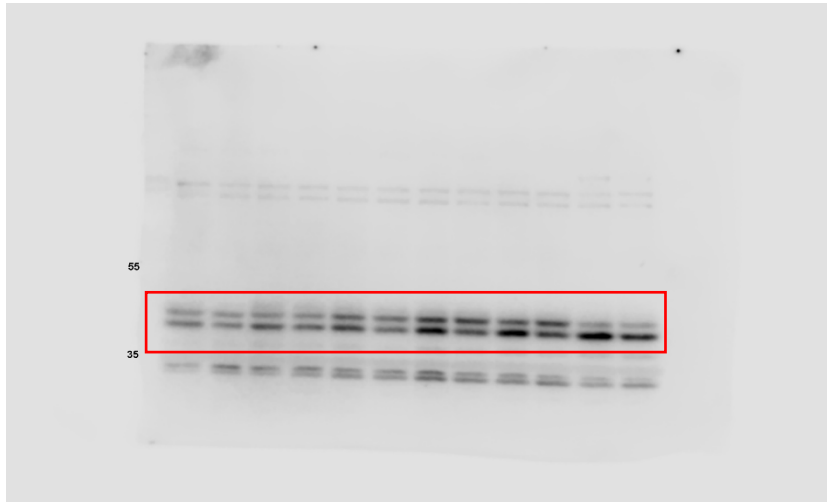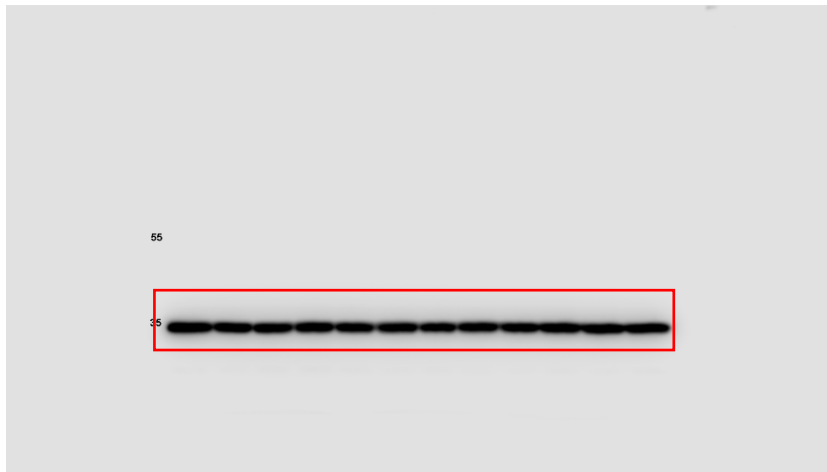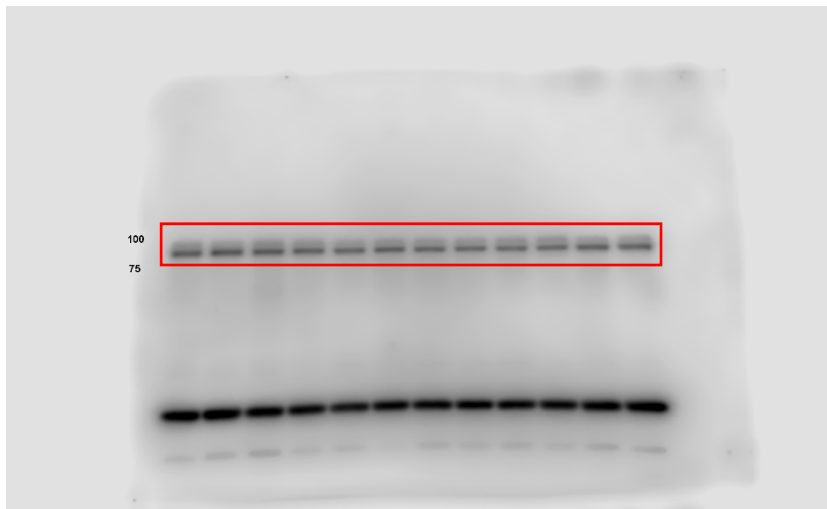

# Figure 6A

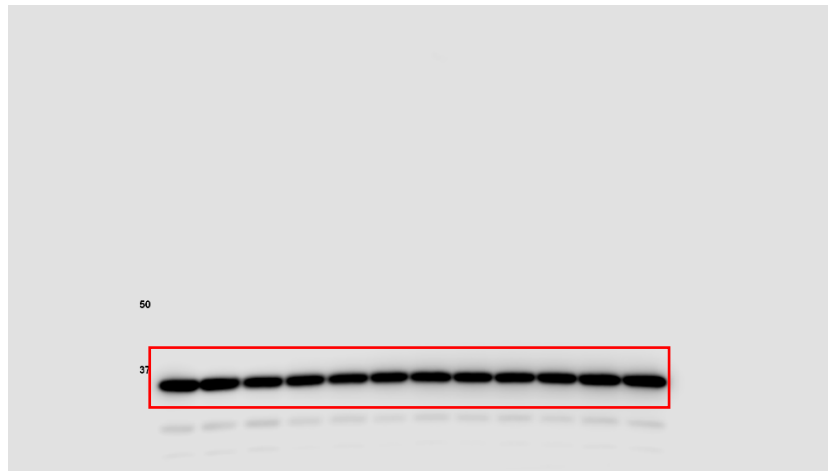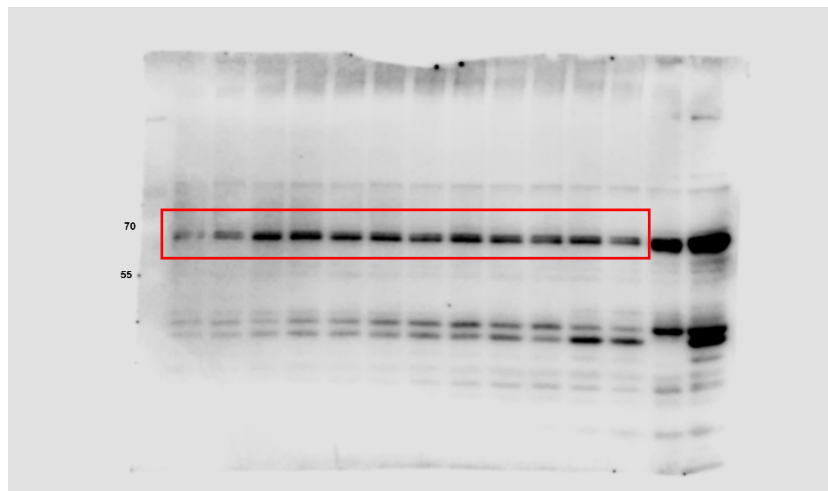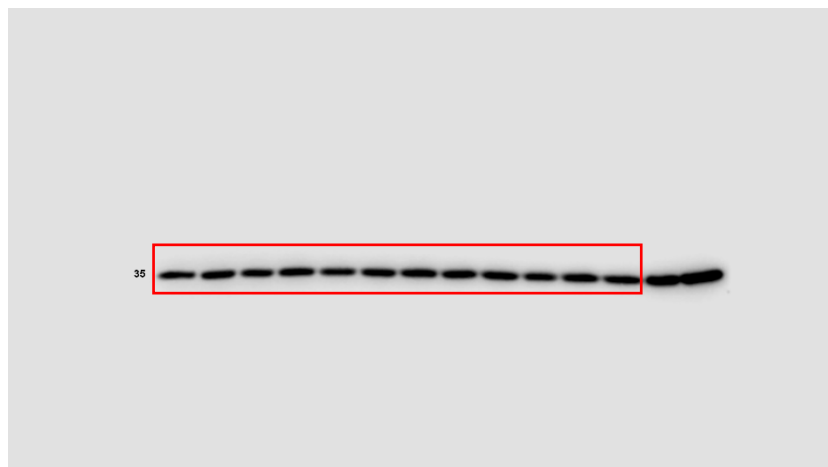

# Figure 6C

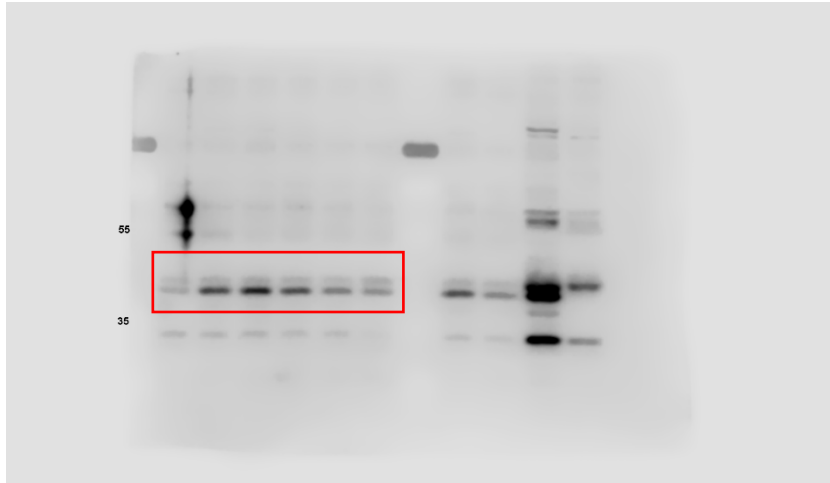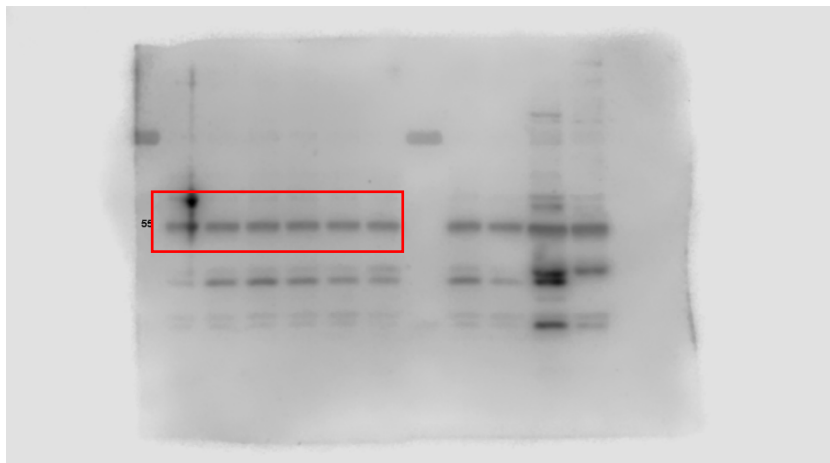

Supplement: Supplementary file 6 [file LSA-2020-00750_SdataF6.pdf]
